# A retrospective analysis of revision framework surgeries for unilateral vocal fold paralysis

**DOI:** 10.1016/j.bjorl.2020.11.016

**Published:** 2020-12-26

**Authors:** Yo Kishimoto, Nao Hiwatashi, Yoshitaka Kawai, Shintaro Fujimura, Tohru Sogami, Yasuyuki Hayashi, Koichi Omori

**Affiliations:** a Kyoto University, Department of Otolaryngology-Head and Neck Surgery, Graduate School of Medicine, Kyoto, Japan; b Kyoto-Katsura Hospital, Department of Otolaryngology, Kyoto, Japan; c Soseikai General Hospital, Department of Otolaryngology, Kyoto, Japan

**Keywords:** Laryngoplasty, Reoperation, Unilateral vocal cords, Paralysis, Voice

## Abstract

**Introduction:**

Revision framework surgeries might be required for unilateral vocal fold paralyses. However, outcomes and indications of revision surgeries have not been adequately documented. For a better understanding of indications for the procedure and to help in achieving better vocal outcomes, we performed a retrospective chart review of patients who underwent revision framework surgeries for unilateral vocal fold paralysis.

**Objectives:**

This study aimed to present clinical features of patients who underwent revision framework surgeries for the treatment of unilateral vocal fold paralysis.

**Methods:**

Of the 149 framework surgeries performed between October 2004 and October 2019, 21 revision framework surgeries were performed in 19 patients. Self-assessments by patients using the voice handicap index-10 questionnaire, and objective aerodynamic and acoustic assessments performed pre- and post-operatively were analyzed using the Wilcoxon’s signed-rank test for paired comparisons.

**Results:**

Undercorrection was indicated as reasons for revision surgeries in all cases. The revision techniques included type I thyroplasty, type IV thyroplasty, and arytenoid adduction, and revision surgeries were completed without any severe complication in all cases. Pre- and post-operative voice handicap index-10 scores were obtained in 12 cases, and other parameters were evaluated in 18 cases. Significant improvements were observed in voice handicap index-10 scores, maximum phonation time, mean flow rate, Current/Direct Current ratio, and pitch perturbation quotient.

**Conclusion:**

Undercorrection was observed in all patients who underwent revision framework surgeries for unilateral vocal fold paralysis, and the initial assessment and planning are thought to be important in order to avoid revision surgeries. Revision surgeries were performed safely in all cases, and significantly improved vocal outcomes were observed, even after multiple procedures. Revision surgery should be considered for patients with unsatisfactory vocal functions after primary framework surgeries for unilateral vocal fold paralysis.

## Introduction

Unilateral vocal fold paralysis occurs following a vagus or recurrent laryngeal nerve injury due to iatrogenic, idiopathic, or other intrinsic or extrinsic causes.^1^ It impairs vocal fold movement resulting in a breathy voice; treatment modalities include injection laryngoplasty and laryngeal framework surgery.

Laryngeal framework surgeries for voice disorders were developed by Isshiki et al. in the 1970s.^2^, ^3^ They are innovative, because the tension on and position of the vocal folds is altered only by changing the form of the framework without directly contacting the vocal folds. Especially, medialization thyroplasty (Type 1 thyroplasty: TP1) and arytenoid adduction (AA) have been widely accepted as effective surgical options for unilateral vocal fold paralysis, and various artificial materials for implants and newer techniques have been introduced.^4^

TP1 and AA are relatively less-invasive surgical treatment modalities, which can be performed under local anesthesia. Phonosurgeons can monitor changes in voice by encouraging the patient to phonate during the surgery, which allows delicate adjustments of glottal closure by changing the size and/or location of implanted materials and the pulling force on arytenoid cartilage.

Although one of the advantages of TP1 and AA, compared to injection thyroplasty, is their persistent effect; sometimes, adequate improvement in vocal function by initial surgery is not obtained, and revision surgery is often required. Further, atrophic changes in the vocal fold mucosa or dislocation of the implanted material can occur with time. In such cases, glottal closure is rendered incomplete, and revision framework surgery is considered.

The concept of revision framework surgery has been well accepted, however, the outcomes and indications of revision TP1 and/or AA have not been adequately documented. For better understanding of the indications for revision procedures and to help in achieving better phonetic outcomes, we performed a retrospective chart review of patients who underwent revision TP1 and/or AA for unilateral vocal fold paralyses.

## Methods

### Patients

Of the 149 framework surgeries performed between October 2004 and October 2019, 19 patients underwent 21 revision laryngeal framework surgeries at the Kyoto University Hospital. Six cases out of 21 cases had prior surgeries in other hospitals and remaining 15 cases had prior surgeries in our hospital.

The patient characteristics and clinical information were retrospectively reviewed. The reviewed data included demographics, etiology of paralysis, prior treatment protocols, and revision techniques used. Written informed consent was obtained from all patients prior to the surgeries, and the study was approved by the institutional review board of the Graduate School of Medicine, Kyoto University (R2387).

### Indications for revision surgery

The revision surgery was considered for patients with unilateral vocal fold paralysis who were not satisfied with the outcomes of prior framework surgery or for those who complained of worsening vocal function over time, after endoscopic confirmation of incomplete glottal closure.

Preoperative endoscopic findings were classified into the following 4 structural characteristics, according to a previous report: anterior glottic incompetence, posterior glottic incompetence, glottic overclosure, and/or decreased phonatory mucosal pliability.^5^

In general, revision TP1 was considered to result in better closure of the anterior part of glottis. According to a previous study,^6^ AA was indicated and performed with or without TP1 in patients with posterior glottic incompetence. In all cases, intraoperative manual cricothyroid approximation was attempted, and if it resulted in better vocal outcomes, thyroplasty type 4 (TP4) was performed simultaneously.

### Assessment of vocal outcomes

Phonatory outcomes before and after framework surgeries were evaluated with self-assessment by the patient using the Voice Handicap Index-10 (VHI-10) questionnaire and with objective aerodynamic and acoustic assessments. Aerodynamic examinations included Maximum Phonation Time (MPT), Mean Flow Rate (MFR), and Alternating Current/Direct Current ratio (AC/DC). For acoustic examinations, the computerized speech lab software (Kay PENTAX, Lincoln Park, NJ) was used to evaluate the Pitch Perturbation Quotient (PPQ), Amplitude Perturbation Quotient (APQ), and Noise to Harmonic Ratio (NHR).

### Statistical analysis

The JMP (SAS Institute Inc., NC) was used for statistical analysis. The Wilcoxon’s signed-rank test was used for paired comparison between the pre- and post-operative vocal outcomes. A *p*-value of less than 0.05 was considered statistically significant.

## Results

### Patient and lesion characteristics

Patient and lesion characteristics are summarized in Table 1, Table 2. Thirteen males underwent 15 revision surgeries, and 6 females underwent 6 revision surgeries. Patient ages at revision surgery ranged from 21 to 83 years (average 63.1 years). All patients presented with unilateral vocal fold paralysis, which was the result of iatrogenic causes in 15 cases. Other causes included idiopathic (4 cases), trauma (1 case), and congenital (1 case). As iatrogenic causes, aortic surgery, thyroidectomy, and neck dissection were identified in 4, 3 and 3 cases, respectively.

**Table 1:** Patient characteristics.

Patient characteristics		
Age		63.1 (21–83) years
Gender	Male	15
	Female	6
Etiology	Iatrogenic	
	Aortic surgery	4
	Thyroidectomy	3
	Neck dissection	3
	Pneumonectomy	2
	Mediastinal surgery	1
	Resection of vagal paraganglioma	1
	Intracranial surgery	1
	Idiopathic	4
	Trauma	1
	Congenital	1

**Table 2:** Lesion characteristics.

Lesion characteristics	
Side of paralysis	
Right	6
Left	15
Endoscopic findings	
Anterior glottic incompetence	10
Posterior glottic incompetence	4
Anterior and posterior glottic incompetence	3
Anterior glottic incompetence and decreased phonatory pliability	2
Anterior and posterior glottic incompetence, decreased phonatory pliability	2

Of the 21 cases, left–sided paralysis were observed 15 cases, and endoscopic examination revealed anterior glottic incompetence, posterior glottic incompetence, and decreased phonatory mucosal pliability in 17 (81.0%), 9 (42.9%), and 4 (19.0%) cases, respectively. No patient demonstrated an overcorrected vocal fold.

### Surgery

Duration between prior framework surgery and revision surgery varied from 1 to 109 months (average 24.1 months). Revision surgery was considered within 6 months after the prior surgery in 11 cases because of insufficient vocal improvements. Revision techniques included TP1, TP4, AA, and their combinations. The procedures are elaborated in Table 3. Of the 21 cases, 16 cases had undergone single prior framework surgeries, and the remaining 5 cases had undergone multiple prior surgeries. All procedures were performed under local anesthesia, and prior/revision TP1 was performed through the thyroid cartilage windows. The prosthesis differs in prior surgeries, however the GORE-TEX® (W. L. Gore & Associates, Inc., AZ) implant was used for TP1 in all revision surgeries.

**Table 3:** Treatment protocols.

Treatment information	
Duration from prior surgery	24.3 (1–109) months
Within 12 months	11
Later than 12 months	10
No. of prior surgery	
1	16
2	3
3	2
Prior surgery	
TP1	15
TP1+TP4	1
TP1+AA	3
TP1+TP4+AA	2
Prosthesis used in prior surgery	
GoreTex	16
Silastic	2
PTFE	2
Unspecified	1
Revison surgery	
TP1	13
AA	5
TP1+TP4	1
TP1+AA	2

In some patients who underwent revision TP1, removal of the previously implanted material was required. Although scar tissue was identified at the implanted site, all implant material was safely removed, and revision TP1 was safely completed in 16 cases. AA was indicated in patients with posterior glottic incompetence, however, the procedure was not performed in 2 cases. One case had undergone prior AA, and severe scarring was observed. The other case presented with improvement in vocal function after revision TP1, and we decided that additional AA would not be required.

Regarding postoperative complications, hematoma was observed in 1 patient who had undergone TP1, which resolved with conservative management. No other major complications such as postoperative bleeding requiring wound exploration, surgical site infection, pyriform sinus fistula, or tear of vocal fold mucosa were observed in any case.

### Vocal outcomes

Pre- and postoperative VHI-10 scores were obtained for 12 cases, and aerodynamic and acoustic examinations were performed in 18 cases. As postoperative examinations were not performed at the same time point in all patients, data obtained between 1 and 6 months after surgery were used for comparison. Significant improvements were observed in VHI-10 scores (*p* = 0.0024), MPT (*p* = 0.0002), MFR (*p* = 0.0208), AC/DC ratio (*p* = 0.0003), and PPQ (*p* = 0.0104); however, changes in APQ (*p* = 0.0814) and NHR (*p* = 0.1324) were not statistically significant (Table 4). Improvements of more than 6 on VHI-10 scores were observed in 9 cases.

**Table 4:** Vocal outcomes.

	Pre	Post	*p*-value
VHI10 (n = 12)	23.6	12.8	0.0024^a^
Aerodynamic (n = 18)			
MPT (sec)	7.6	10.7	0.0002^a^
MFR (mL/s)	478.0	280.6	0.0208^a^
AC/DC	23.2	37.2	0.0003^a^
Acoustic (n = 18)			
APQ	5.0	3.2	0.0814
PPQ	2.67	1.2	0.0104^a^
NHR	0.18	0.13	0.1324

VHI, Voice handicap index; MPT, Maximam phonation time; MFR, Mean flow rate; AC/DC, AC/DC ratio; APQ, Amplitude perturbation quotient; PPQ, Pitch perturbation quotient; NHR, Noise to harmonic ratio.

a *p* < 0.05.

## Discussion

In this study, we retrospectively reviewed cases treated with revision framework surgeries for unilateral vocal fold paralyses, and our results suggested that revision framework surgeries are a safe and promising option for patients exhibiting unsatisfactory results with prior framework surgeries.

Framework surgeries such as TP1 and/or AA for unilateral vocal fold paralysis have become widely accepted, and the favorable success rate of the procedure has been well-documented.^7^ However, according to a national survey in the United States, unchanged and worsened voice after medialization laryngoplasty were observed in 3% and 1% patients, respectively, and the revision rate accounted for 6%.^7^ Thus, revision framework surgeries are rare but are occasionally required.

In our case series, 15 of the 21 cases had iatrogenic causes such as thyroidectomy, aortic surgery, or neck dissection, and this finding is similar to previous studies of primary or revision framework surgeries.^5^, ^8^, ^9^ Regarding the causes for revision surgeries, endoscopic examination revealed anterior and/or posterior glottic incompetence in all patients, and no overcorrection was observed. Cohen et al. reported that the most common problem in glottal closure was undercorrection,^10^ and this is consistent with the results of our study.

Revision surgery was considered in early postoperative period in 11 cases, and inadequate correction in prior surgery was indicated. In fact, seven cases that underwent AA complained of hoarseness just after the prior surgeries, and revision surgeries were suggested within 6 months after the prior surgeries. AA should be considered in the prior surgery in these cases, and the initial assessment and planning are thought to be important for the better outcomes.

Regarding the prostheses, GORE-TEX or PTFE were used in 18 of 21 cases in prior surgeries. Higher prevalence of under correction with sheet-like material has been reported,^11^ and the type of materials might be one of the reasons for the failure in prior surgeries. Because Isshiki has recommended overcorrection in the adjustment procedure,^12^ we demonstrated intraoperative flexible laryngoscopy and preferred overcorrection in revision TP1 using GORE-TEX, and this might contribute to good outcomes in our case series.

During revision surgeries, post-operative scars from prior surgeries render the procedures more difficult compared to primary surgeries.^13^ Sometimes, it is not easy to locate the implant and thyroplasty window of prior surgery. In our case series, implants were easily identified and were removed without any severe damage to the surrounding tissues, and revision surgeries were completed without interruption or cessation of the procedures except for one case. Although two patients with prior AA were expected to have revision AA in our cases, one patient did not undergo revision AA because of severe scar formation. However, Matsushima et al. have reported successful revision AA in patients with prior AA,^14^ and also we have completed revision AA in one case. Thus, laryngeal framework surgery was thought to be revisable and reversible, if performed carefully.

Complications of TP1 and/or AA include postoperative bleeding, hematoma formation, laryngeal edema, laryngeal or pharyngeal fistula, and laryngeal granulation, and their incidence has been reported to be 8.5%–16% in primary surgery.^15^, ^16^ During revision surgeries, vocal fold mucosa or pyriform sinus mucosa could be easily torn, because fibrous changes render the tissues stiff. Thus, rates of complications could be higher in revision surgeries. However, in our case series, only one patient presented with postoperative hematoma, and no other complications were observed. As shown in Table 5, previous retrospective studies have not demonstrated high complication rates, and this shows postoperative complications can be avoided with careful and cautious procedure performance.

**Table 5:** Summary of published case series of revision framework surgeries.

Article	Operations	Patients	Procedures	Outcome measures	Post-operative improvement	Complications
Maragos^15^	61	48	Type I, II, III, IV, AA, AF	Jitter, Shimmer, NHR	Jitter (Female), NHR	Not listed
Woo et al.^11^	27	20	Type I, IA, AA, IR	MPT, MFR, F0, Jitter, Shimmer, NHR	MFR	Not listed
Cohen et al.^10^	22	16	Type I, IA, AA, IR	GCI, VRS	GCI	None
Parker et al.^5^	48	39	Type I, CTS, Arytenopexy	VRQOL, MFR, Air pressure, F0, Jitter, Shimmer, NHR	VRQOL, F0 (Female), Jitter, NHR	Hematoma (2), Cellulitis (1), Subcutaneous emphysema (1)

AA, Arytenoid adduction; AF, Arytenoid fixation; IA, Injection augmentation; IR, Implant removal; CTS, Cricothyroid subluxation; NHR, Noise to harmonic ratio; MPT, Maximam phonation time; MFR, Mean flow rate; F0, Fundamental frequency; GCI, Glottal closure index; VRS, Voice rating scale; VRQOL, Voice related quality of life.

Regarding vocal outcomes, significant improvements were observed in VHI10, MPT, MFR, AC/DC ratio, and PPQ, but not in APQ and NHR. In previous studies, vocal outcomes have been evaluated with varied parameters, and thus, it is difficult to compare our results with the results of other studies. Some improvements have been observed in all studies, and it was suggested that revision framework surgeries contribute to improvement in vocal functions in patients with unilateral vocal fold paralyses.^5^, ^10^, ^13^, ^17^ Although Misono suggested that a difference of 6 in VHI-10 score represents a minimum significant difference, 3 of 12 cases demonstrated improvements of less than 6 in VHI-10 scores.^18^ Further, while significant improvements in acoustic parameters after primary framework surgeries have been documented, significant improvements in APQ have not been demonstrated in any previous study of revision surgeries. Alterations in the viscoelastic properties of vocal fold mucosae and muscles caused by multiple surgical procedures could limit the acoustic outcomes of framework surgeries.

One of the limitations of this study was its retrospective design. The sample size was small, and vocal outcomes were not evaluated at the same time point in all cases, and it is difficult to elucidate reasons of failure in prior surgeries or indications of revision procedures. However, at least we confirmed that revision surgeries were required because of undercorrection in all cases and initial assessment and planning were thought to be important. Also, we observed that revision framework surgery for unilateral vocal fold paralysis is safe and could contribute to improvement in vocal functions. Further prospective studies with larger sample sizes are warranted to clarify the indications and limitations of revision framework surgeries for unilateral vocal fold paralysis.

## Conclusion

This retrospective chart review of patients who underwent revision TP1 and/or AA for unilateral vocal fold paralysis showed undercorrection in all cases, and determined the importance of the initial assessment and planning to avoid revision surgeries.

Revision framework surgeries for unilateral vocal fold paralyses were completed safely, and significant improvements in vocal outcomes were confirmed even after multiple procedures. Revision surgery is a promising option and should be considered for patients with unsatisfactory vocal functions after primary framework surgeries for unilateral vocal fold paralysis.

## Conflicts of interest

The authors declare no conflicts of interest.
